# A review of trisomy X (47,XXX)

**DOI:** 10.1186/1750-1172-5-8

**Published:** 2010-05-11

**Authors:** Nicole R Tartaglia, Susan Howell, Ashley Sutherland, Rebecca Wilson, Lennie Wilson

**Affiliations:** 1 Department of Pediatrics, University of Colorado Denver School of Medicine, 13123 East 16th Ave, Aurora, Colorado, 80045, USA; 2 Child Development Unit, The Children's Hospital, 13123 East 16th Ave B140, Aurora, Colorado, 80045, USA; 3 Psychiatry and Behavioral Services, Montefiore Medical Center, 111 East 210th Street, Bronx, New York, 10467, USA

## Abstract

Trisomy X is a sex chromosome anomaly with a variable phenotype caused by the presence of an extra X chromosome in females (47,XXX instead of 46,XX). It is the most common female chromosomal abnormality, occurring in approximately 1 in 1,000 female births. As some individuals are only mildly affected or asymptomatic, it is estimated that only 10% of individuals with trisomy X are actually diagnosed. The most common physical features include tall stature, epicanthal folds, hypotonia and clinodactyly. Seizures, renal and genitourinary abnormalities, and premature ovarian failure (POF) can also be associated findings. Children with trisomy X have higher rates of motor and speech delays, with an increased risk of cognitive deficits and learning disabilities in the school-age years. Psychological features including attention deficits, mood disorders (anxiety and depression), and other psychological disorders are also more common than in the general population. Trisomy X most commonly occurs as a result of nondisjunction during meiosis, although postzygotic nondisjunction occurs in approximately 20% of cases. The risk of trisomy X increases with advanced maternal age. The phenotype in trisomy X is hypothesized to result from overexpression of genes that escape X-inactivation, but genotype-phenotype relationships remain to be defined. Diagnosis during the prenatal period by amniocentesis or chorionic villi sampling is common. Indications for postnatal diagnoses most commonly include developmental delays or hypotonia, learning disabilities, emotional or behavioral difficulties, or POF. Differential diagnosis prior to definitive karyotype results includes fragile X, tetrasomy X, pentasomy X, and Turner syndrome mosaicism. Genetic counseling is recommended. Patients diagnosed in the prenatal period should be followed closely for developmental delays so that early intervention therapies can be implemented as needed. School-age children and adolescents benefit from a psychological evaluation with an emphasis on identifying and developing an intervention plan for problems in cognitive/academic skills, language, and/or social-emotional development. Adolescents and adult women presenting with late menarche, menstrual irregularities, or fertility problems should be evaluated for POF. Patients should be referred to support organizations to receive individual and family support. The prognosis is variable, depending on the severity of the manifestations and on the quality and timing of treatment.

## Background/Definition

Trisomy X (47,XXX) is a sex chromosome aneuploidy condition in which females have an extra X chromosome, compared to the 46,XX karyotype in typical females. It was first described in 1959 in a 35-year-old woman with normal intellectual abilities who presented with secondary amenorrhea at 19 years of age [1]. Since the initial description, only several hundred cases have been described, identifying a variety of associated developmental, psychological, and medical features. Most of the background literature on trisomy X comes from longitudinal prospective studies of females identified by newborn screening and followed into young adulthood. These studies were conducted in the 1970's and 80's at multiple centers across the U.S., Canada, and the U.K. [2-5]. While newborn screening studies have demonstrated that the incidence of trisomy X is approximately 1/1000 female births, only approximately 10% of cases are ascertained clinically. There is considerable variation in the phenotype, with some individuals very mildly affected and others with more significant physical and psychological features. This manuscript reviews the current literature available describing features associated with trisomy X, with recognition that much of the literature is based on small sample sizes and clinical ascertainment of patients, and does not likely represent the full spectrum of females with trisomy X. However, review of the current knowledge is necessary to provide a summary of background and treatment recommendations for patients and professionals, and to highlight the many areas of need for additional research in trisomy X.

## Disease Names/Synonyms

Trisomy X is also commonly known as:

47,XXX

Triple X, or

Triplo-X

## Epidemiology

Originally described as the "superfemale" in 1959, trisomy X occurs in approximately 1 in 1,000 female births, however, it is estimated that only approximately 10% of cases are diagnosed [6]. In identified cases, diagnosis occurs through prenatal amniocentesis or chorionic villi sampling (CVS), or in the postnatal period through a standard karyotype test or chromosome analysis performed for hypotonia, developmental delays, physical features, or cognitive/behavioral difficulties. Although nonmosaic 47,XXX karyotypes are the most frequent, mosaicism occurs in approximately 10% of cases and can occur in many combinations such as 46,XX/47,XXX or 47,XXX/48,XXXX, or in combinations including Turner syndrome cell lines such as 45,X/47,XXX or 45,X/46,XX/47,XXX[6].

## Clinical Description

### A. Physical Characteristics

**Figure 1:**
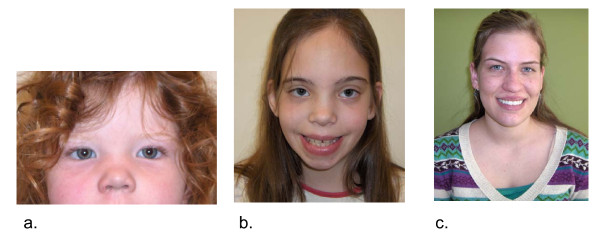
**Variable facial features in girls with trisomy X**. (a) Epicanthal folds and hypertelorism in 2 year old girl, (b) Hypertelorism in 9 year old girl, (c) Lack of dysmorphic features in a 19 year old girl with trisomy X.

Significant facial dysmorphology or striking physical features are not commonly associated with 47,XXX, however, minor physical findings can be present in some individuals including epicanthal folds, hypertelorism, upslanting palpebral fissures, clinodactyly, overlapping digits, pes planus, and pectus excavatum. Hypotonia and joint hyperextensibility may also be present [2,7]. Please see Figure 1 for photographs of girls with trisomy X, and Table 1 for a summary of physical and medical features.

**Table 1:** Physical and Medical Features Associated with Trisomy X

Feature	**Estimated frequency based on current available data **[10,19,22,33,44]
Tall stature >75^th ^percentile	80-89%

Epicanthal folds	32-46%

Clinodactyly	42-65%

Hypotonia in infancy	55-71%

Genitourinary malformations	5-16%

Seizure disorder	11-15%

Intention tremor	6-20%

Congenital hip dysplasia	2-12%

Constipation/Abdominal pains	12-45%

Premature ovarian failure	unknown

Length and weight at birth is usually normal for gestational age, however, stature typically increases in early childhood, and by adolescence most girls with 47,XXX are at or above the 75^th ^percentile for height [2]. A few cases have been ascertained due to tall stature [8], and current evaluation of tall stature in females should include karyotype analysis to evaluate for 47,XXX. Cases of short stature have also been described (unrelated to a known 45,X mosaicism), and one prospective study identified a subgroup of 47,XXX girls with heights below the 50^th ^percentile [9]. Body segment proportions typically show long legs, with a short sitting height [10]. Studies of bone age have shown no significant differences from 46,XX females [11]. The average head circumference is below the 50^th ^percentile, however, there is a lot of individual variation. Microcephaly (<5^th ^percentile) is rare [12,13].

### B. Clinical characteristics

Although major medical problems are not present in most cases, other medical problems may be associated with trisomy X. The most common are genitourinary abnormalities, ranging from unilateral kidney and renal dysplasia to ovarian malformations [14]. Congenital heart defects have also been described including cases of atrial and ventricular septal defects, pulmonic stenosis, and aortic coarctation [15-17]. Studies describing seizure disorders and EEG abnormalities in trisomy X vary from 0 to 65% depending on the cohort studied and means of ascertainment, however, in the largest cohorts clinical seizures are present in approximately 15% of cases. Seizure subtypes including absence, partial, and generalized seizures have been described, with good responses to standard anticonvulsant treatments [18-21]. Gastrointestinal problems, including constipation and abdominal pain, are also common concerns [2,22].

Pubertal onset and sexual development are usually normal in trisomy X, however, there have been cases of ovarian or uterine dysgenesis described in children and young adults with trisomy X. Premature ovarian failure (POF) is a condition in which the ovarian functions of hormone production and oocyte (egg) development become impaired before the typical age for menopause. There are multiple case reports of women with trisomy X found to have POF, with endocrine findings of hypergonadotropic hypogonadism. The ages of these cases have ranged from 19 to 40 years [1,23,24]. Studies on the prevalence of POF in adolescents or adults with trisomy X have not yet been performed. One study that performed genetic screening on women presenting with POF identified trisomy X in 3% of cases [25]. In trisomy X, a large percentage of the reported cases of POF have also been associated with other autoimmune diseases [23,26,27], including autoimmune thyroid disorder [25].

Precocious puberty has also been described but is not a typical finding. There have been no direct studies of fertility in trisomy X, however, many reports of successful pregnancies have been described, and fertility is likely normal in most cases unless complicated by a genitourinary malformation or POF as described above [2]. There is a significant need for more research in this area in the trisomy X population.

### c. Developmental and Psychological Characteristics

There is significant variability in the developmental and psychological features of children and adults with trisomy X, ranging from those with minimal involvement to those with clinically significant problems requiring comprehensive intervention services. Thus, individual evaluation of developmental and psychological problems known to be associated with trisomy X is important in each individual.

Infants and toddlers are at increased risk for early developmental delays, especially in speech-language development and motor development related to hypotonia. Average age at walking independently is 16.2 months (range 11-22 months), and for first words is 18.5 months (range 12 - 40 months) [2]. Prospective studies comparing girls with trisomy X at 24 months of age to sibling controls show impairments in speech and language development. Expressive language may be more impaired than receptive language, with a pattern described as developmental dyspraxia in some patients. However, other patients show impairments in both expressive and receptive language [28]. Speech and language deficits may continue throughout childhood into adulthood, with higher level language difficulties including problems with language processing, verbal fluency, language comprehension, and pragmatic language in some patients [2,28,29].

**Figure 2:**
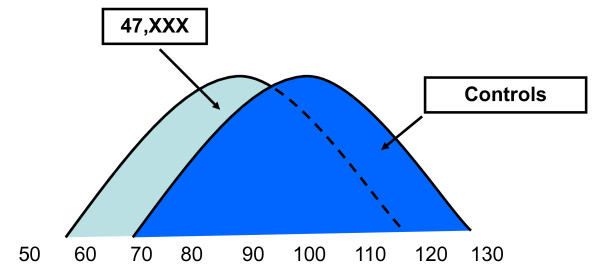
**Estimated Full-Scale IQ (FSIQ) distribution for females with 47,XXX compared to controls**. In 47,XXX, there is a wide variation in IQ with the normal curve shifted to the left with the mean FSIQ at 85-90. The majority of females with 47,XXX have a FSIQ in the normal range, and many are above average compared to the general population. However, due to the shift of the curve, a larger percentage of females with 47,XXX fall in the intellectual disability range compared to controls. Adapted and reprinted with permission from Bender et. al. 1986 [42].

Studies on cognitive abilities in trisomy X also show a wide range of cognitive skills, with full scale IQ's ranging from 55-115 across various studies [28-34]. While there are clearly many girls with trisomy X with cognitive skills in the average to above average range, cognitive deficits and learning disabilities are more common than in the general population and when compared to sibling controls. IQ subscales most commonly reveal deficits in verbal IQ compared to nonverbal/performance IQ, however, many patients with trisomy X have cognitive deficits across both verbal and nonverbal domains [3,29,32,35,36]. While cognitive deficits in the intellectual disability (mental retardation) range are rare, intellectual disability is more common than in the general population with the mean full scale IQ at 85-90 and approximately 5-10% with intellectual disability [37]. See Figure 2.

Motor skill deficits may also be present. Walking may be delayed, and decreased muscle tone and lack of coordination are often clinically significant. An extensive motor study of 10 children with trisomy X showed ongoing motor planning difficulties and overall weaknesses in motor skills and motor coordination, along with gait abnormalities and poor joint stability [2,38].

Attentional problems, poor executive function, and decreased adaptive functioning skills may also impact educational and home functioning. Attention deficit hyperactivity disorder (ADHD) is present in 25-35% of cases, with symptoms of inattention, distractibility, and poor organization typically more significant than hyperactivity [29,39].

There is a paucity of research on mental health problems in trisomy X, however, increased rates of anxiety, depression/dysthymia and adjustment disorders have been described in previous studies [2,40]. Anxiety concerns are mostly related to social avoidance, generalized anxiety and separation anxiety, and can present in the early school age years or in adolescence. Childhood anxiety and language weaknesses are a difficult combination for affected children as the demanding verbal environments often found in school settings can exacerbate anxiety and result in behavioral difficulties. Language deficits may also impact social adjustment in some children when they have difficulty communicating with playmates and when self-expression is limited in older children and adolescents. Social immaturity relative to peers may be present, and this, along with cognitive and executive function impairments, can make some girls with trisomy X vulnerable to social pressures from peers and victimization. Other mental health disorders (including adjustment disorders, mood disorders and psychotic disorders) have been described in case series and reports [2,41-43], and comprehensive studies evaluating features of these disorders in the trisomy X population are needed. A comprehensive review of trisomy X literature with an emphasis on mental health has recently been published by Otter et al. [44]. Again, the variability in the phenotype needs to be emphasized, since many females with trisomy X have minimal cognitive, social, or emotional difficulties.

### d. Neuroimaging studies

Since the longitudinal studies of the 1970's and 80's of females with trisomy X followed into adulthood, there have been very few additional research studies focused exclusively on this genetic disorder. A neuroimaging study conducted in 2002 in 10 girls with trisomy X from the original longitudinal cohort in Denver (mean age 29.1 years) showed that whole brain volumes were significantly reduced compared to controls, and a slight reduction in the size of the amygdala was also noted [45]. Another MRI study of 12 girls with trisomy X (mean age 21.6) from a birth cohort in the UK confirmed smaller whole brain volumes, and there was a significant correlation between smaller brain volumes and lower cognitive scores on two measures (the national adult reading test/NART and the Quick test) [46]. This study also reported findings of white matter "high intensity foci" (HIS) in 27% of females with trisomy X, similar to those seen in other sex chromosome aneuploidy groups in their study (XXY and XYY), and in other sex chromosome aneuploidy populations such as 48,XXYY and 49,XXXXY [47,48]. The significance or neuropathological findings of these white matter abnormalities are not yet known, however, they suggest that gene dosage effects from sex chromosome genes affect white matter development.

## Genetics

In typical 46,XX females, only one X chromosome in each cell is genetically active and the other is inactivated through DNA methylation and the accumulation of a histone variant throughout the chromosome [49]. X-inactivation occurs early in blastogenesis and is controlled by the X chromosome inactivation center (XIC), which counts the X chromosomes present and randomly inactivates all but one X chromosome per diploid set. The randomly selected X chromosome silences itself by expressing the *XIST *and *Tsix *genes. However, particular segments of the X chromosome, known as the pseudoautosomal regions (PAR1 and PAR2), have Y chromosome homologs and therefore are not inactivated and remain genetically active [50,51]. Approximately 5-10% of additional genes on the X chromosome outside the PAR regions also escape X-inactivation. Thus, in trisomy X, two of the three X chromosomes are inactivated, however, genes in the PAR regions and other genes that escape X-inactivation are expressed from the three X chromosomes. It is hypothesized that the phenotypic abnormalities associated with trisomy X result from overexpression of these genes on the X chromosome that escape X-inactivation [30,52,53]. While there is some microarray evidence of overexpression of X-chromosome genes in cells lines with supernumerary X chromosomes [54], the specific genes involved in the phenotype of trisomy X and other sex chromosome aneuploidies have not been identified. One exception is the *SHOX *gene, which escapes X-inactivation and is associated with the short stature seen in Turner syndrome and the tall stature in supernumerary sex chromosome aneuploidy conditions [55,56].

## Etiology

Trisomy X occurs from a nondisjunction event, in which the X chromosomes fail to properly separate during cell division either during gametogenesis (resulting in a trisomic conceptus), or after conception (known as post-zygotic nondisjunction). Studies of the parental origin of the additional X chromosome in trisomy X demonstrated that 58-63% of cases were derived from maternal meiosis I errors, 16-17.4% were derived from maternal meiosis II errors and 18-19.6% were derived from post-zygotic nondisjunction [57,58].

Similar to other trisomies, trisomy X has been shown to have a statistically significant correlation with advancing maternal age, as the likelihood of nondisjunction events during meiosis increases with increasing maternal age. In one study, mosaic trisomy X (such as 45,X/47,XXX) did not demonstrate a significant age-dependent correlation, suggesting that cases of mosaicism may result from a post-zygotic nondisjunction event [59]. However, cases of 46,XX/47,XXX and 45,X/47,XXX mosaicism may also result from a post-zygotic trisomy rescue.

## Diagnosis

Karyotype analysis of peripheral blood is the most standard test used to make the diagnosis. Prenatal amniocentesis or CVS also identify a percentage of patients with trisomy X, however, confirmation studies are recommended after birth via FISH to study 50+ cells in order to evaluate for mosaicism. It is also important to identify mosaicism with a Turner syndrome (45,X) cell line in order to determine appropriate medical evaluations and treatments needed for Turner syndrome.

The physical and psychological manifestations of trisomy X are variable, and a karyotype should be considered in females presenting with:

▪ Developmental delays (speech and/or motor)

▪ Hypotonia

▪ Hypertelorism/Epicanthal folds/Clinodactyly

▪ Tall stature

▪ Premature Ovarian Failure/Primary Ovarian Insufficiency

▪ Learning Disability/Intellectual Disability

▪ Attention deficits/Attention Deficit/Hyperactivity Disorder (ADHD)

▪ Anxiety, Mood disorders, or other psychiatric symptoms

## Differential Diagnosis

Developmental and behavioral features in trisomy X can be similar to those seen in females with fragile X syndrome. Females suspected of having fragile X with a negative fragile X test should have a karyotype completed to evaluate for trisomy X [16].

Tetrasomy X and pentasomy X syndromes share most features of trisomy X, however, they are usually associated with more significant developmental delays, dysmorphic features (absent in trisomy X), and congenital malformations compared to trisomy X [16,53]. Females with pentasomy X typically have short stature [56].

Due to features in the newborn period such as hypotonia, hypertelorism and epicanthal folds, some patients with trisomy X are ascertained by karyotype performed due to suspicion for trisomy 21 or trisomy 21 mosaicism.

Other genetic conditions associated with tall stature could also be considered depending on the clinical presentation, such as Marfan syndrome (long limbs, hyperextensibility), and the Sotos' and Beckwith-Weidemann syndromes (cognitive impairments).

Adolescents or adult females presenting with POF should be tested for trisomy X, Turner syndrome, and the fragile X premutation, and should have further evaluation to identify other possible medical causes of POF.

Females with trisomy X may first present with a clinical picture that leads to a diagnosis of a neurodevelopmental disorder such as speech-language disorder, learning disability, ADHD, autism spectrum disorder, or anxiety/mood disorder. Individuals with these diagnoses should be further evaluated medically to determine if testing for trisomy X or other medical conditions is indicated.

The differential diagnosis is eliminated after results of a karyotype analysis show trisomy X (47,XXX), unless there are significant impairments (moderate or severe intellectual disability), congenital malformations, or medical problems not consistent with the trisomy X phenotype. In these cases, additional genetic and medical evaluation is warranted to rule out other disorders, since these may co-exist with trisomy X due to the high incidence of 1:1000 births.

Also, a 47,XXX cell line is present in 5-15% of females with Turner syndrome. Karyotype testing of females presenting with short stature and a Turner syndrome phenotype have shown findings of nonmosaic 47,XXX in blood lymphocytes, however, genetic testing of another tissue such as skin biopsy or buccal cells identified 45,X mosaicism [59]. Thus, individuals found to have trisomy X with a Turner syndrome phenotype should have a second tissue type analyzed by cytogenetic or FISH studies to further evaluate for a 45,X cell line since this changes treatment recommendations.

## Genetic Counseling For Trisomy X

Genetic counseling for prenatally diagnosed cases of trisomy X should address the medical, developmental, and psychological manifestations of the condition as outlined in this review. As noted, there is significant variability in developmental delays, learning disabilities and psychological characteristics in trisomy X and it is not yet possible to predetermine which child will exhibit any or all of these concerns. Couples with a recent diagnosis may be eager to search the internet for information about trisomy X and they should be cautioned about the excessive inaccurate and biased information they may find. Couples should be informed of the high frequency of trisomy X (1 per 1000 female births) and that most girls go undiagnosed, in order to support them in understanding and accepting that their diagnosis is not an isolated case with a predetermined outcome [46]. Some couples have found it helpful to talk with other parents of trisomy X children (resource: KS&A (Knowledge, Support & Action), UNIQUE, Triplo-X support group) [60-62]. Couples should be informed that the occurrence of trisomy X is due to a random event, that there is nothing that they did to cause or prevent the occurrence. It is important for parents to appreciate the significant role of other genes inherited, stressing that the child's prognosis is relative to their entire genetic makeup, as well as the impact of environmental factors. In addition, a study of females with trisomy X by Robinson et al. demonstrated that females diagnosed in the prenatal period had better developmental and educational outcomes and more typical peer relationships in comparison to girls diagnosed in the postnatal period [63]. However, this study may be biased as it was based on a small sample of patients and did not control for several environmental influences, such as socioeconomic status and family support.

Couples should be informed that fetal survival rate is good, with 99% surviving to term following diagnosis after amniocentesis [64,65]. It is not routine to karyotype parents of girls with 47,XXX as recurrence risk is estimated to be <1% [66-68]. Similar to other aneuploidies, a significant maternal age effect has been demonstrated with an increasing risk specific to trisomy X of 1/2500 live births at a maternal age of 33 to 1/450 live births at a maternal age of 43 [69]. Studies reporting elective termination rates following a prenatal diagnosis vary by location of the study, with the most recent reports from the U.S. and France ranging from 25-40% [70,71]. These rates are lower compared to other sex chromosome aneuploidy conditions due to the lack of association with infertility and other serious medical problems, and the broad phenotypic variability. For couples who choose to continue an affected pregnancy, it is recommended that the genetic counselor assist the family in identifying community resources for developmental assessments and early intervention services due to the increased risk for developmental delays as outlined in this review. Follow-up postnatal karyotyping is also recommended due to mosaicism in approximately 10% of cases and the potential impact for the medical and developmental management of the child [6].

When discussing the diagnosis with the child, it is important to use terminology that is straightforward and developmentally appropriate. Young children should be given simple, age-appropriate information on a fairly regular basis allowing them to grow, cope and adjust to the information over time. With a young child, it is important to dispel fears of the disorder being lethal or contagious, and to explain that it is not her parents' fault. Discussions with adolescents and young adult children should be straightforward with full disclosure. Use of the term "sex" or "sex chromosome" should be used with caution, as children and adolescents can confuse the diagnosis with their sexuality or misconstrue the diagnosis as having a "sex abnormality." Open communication should be encouraged and questions addressed directly. If the parent does not feel well-informed or prepared to answer questions, seeking input from professionals, such as a physician or genetic counselor, is recommended.

Genetic counseling for adult patients should additionally address potential reproductive issues, specifically POF and the risk of transmission. While fertility in women with trisomy X is generally considered normal, there is an increased risk for POF as noted within this review, which may be important to consider in family planning. Furthermore, patients should be counseled that the transmission of X aneuploidy (extra or missing X chromosomes) from women with trisomy X is rare, although it has been reported [68]. Although some reports support a <5% increased risk for chromosomally abnormal offspring [3,72], more recent figures support that <1% may be more accurate. It is important that this risk be presented independently of the risks due to maternal age. These transmission risks apply only to women with non-mosaic 47,XXX, as mosaicism may increase the risk of X aneuploidy and potential outcomes, so each scenario should be considered individually [3,72,73].

## Management

Evaluation and treatment recommendations depend on the age of the patient and severity of the phenotype, however, all individuals should undergo a medical history and physical examination with an emphasis on features requiring monitoring and intervention as outlined in this review. Infants and children with trisomy X should undergo evaluation for the psychological and medical features of the disorder. In infants and young children, a renal ultrasound and cardiac evaluation should be obtained. Constipation should be treated as needed. Medical history should include questions regarding staring spells or atypical movements, since seizure disorders and electroencephalogram (EEG) abnormalities can be present in females with trisomy X and may present as partial or absence seizures. In these cases, EEG studies should be performed to rule out possible seizure activity.

Adolescents and adult women presenting with late menarche, menstrual irregularities, or problems with fertility should be evaluated by an endocrinologist or gynecologist for hormonal abnormalities that may signal ovarian insufficiency which can be associated with trisomy X. Other autoimmune problems including thyroid problems should also be considered.

A comprehensive developmental evaluation is important for newly diagnosed infants and young children, and between 6-12 months of age for infants diagnosed in the prenatal period. The assessment should include special emphasis on language, motor, and social development. Early developmental stimulation, speech therapy, occupational therapy and/or physical therapy should be considered, especially if assessment results show scores within the delayed or borderline range. For school-age children and adolescents, a multidisciplinary assessment, including evaluation with a child psychologist (for learning disabilities, social/emotional problems, and adaptive functioning assessment), as well as speech/language assessment and occupational therapy assessment, is important in order to identify strengths and weaknesses and to help develop educational supports and behavioral interventions. Common problems including learning disabilities, speech-language disorders (including apraxia of speech), ADHD with predominantly inattentive symptoms, executive dysfunction, anxiety disorders, social difficulties, and other mental health problems should be considered and treated if problematic. Consultation with a developmental pediatrician, psychiatrist, or neurologist is important in females with trisomy X who have associated ADHD, anxiety, and other mental health problems to discuss possible behavioral and/or medication treatments. In females with trisomy X who have these conditions, medication treatments are the same as for the general population, however, low starting doses are recommended due to the more complex neurodevelopmental involvement in trisomy X.

It is important for evaluators to recognize that behavioral symptoms related to learning disabilities, ADHD, language comprehension deficits, and anxiety may have significant overlap, and thus consideration and treatment of all the comorbidities are important in developing a treatment plan. Psychological therapy and counselling can be effective as a part of the treatment plan if needed, however, may need to be modified based on the receptive-expressive language and cognitive abilities of the patient. If present, developmental concerns and educational struggles should be addressed aggressively instead of taking a "wait and see" approach, since they are unlikely to improve or "catch up" without targeted interventions, and delaying treatment will lead to poorer outcomes. Assessment and documentation of adaptive functioning (life skills) in domains including self-care, communication, social, community use, safety, and self-direction is important to identify strengths and weaknesses in these areas. A subset of females with trisomy X has borderline cognitive abilities or learning disabilities with adaptive functioning in the disability range, and in this group adaptive functioning assessment is important to support the need for community services and disability supports through adulthood.

## Family Support

Family support can be a very important part of treatment, especially for families of girls with more severe medical or psychological features of trisomy X. Support organizations include KS&A in the U.S., UNIQUE in Europe, and a Triplo-X internet-based support group supporting families internationally [60-62]. Families of children and young adults with trisomy X and associated developmental delays or mental health problems should also be encouraged to seek out local support groups for general developmental disabilities or mental health problems since these organizations have access to resources in the local area for the family.

## Prognosis

The prognosis of trisomy X is variable, with some individuals doing extremely well with minimal manifestations of the disorder, and others with more significant cognitive and psychological involvement as described above. Outcomes of those diagnosed in the prenatal period have been found to be better than those of patients described in the prospective studies (birth cohorts) and than those in case reports of girls identified after birth ascertained due to developmental delays [64]. Girls with 46,XX/47,XXX mosaicism also have improved outcomes compared to those with 100% 47,XXX [37].

## Unresolved Questions

There are many unresolved questions in trisomy X, as this genetic disorder has received very little attention by scientists or clinicians since the completion of the prospective, descriptive studies of the 1970's and 80's. Additional research on the pathophysiology and genetic mechanisms involved in the associated medical problems (such as seizures and POF) is needed. Elucidation of the specific genes, gene pathways, and genetic mechanisms involved in the phenotype and phenotypic variability will help to understand the pathophysiology, improve genetic counseling, and perhaps lead to targeted treatments in the future. Clinical studies are also needed to further characterize the psychological features and neurodevelopmental disorders, and to study specific interventions for developmental delays, learning disabilities, and psychiatric problems in this population, in order to guide parents, educators, and mental health professionals.

## List of Abbreviations

POF: Premature ovarian failure; IQ: Intelligence quotient; FSIQ: Full scale intelligence quotient; VIQ: Verbal IQ; PIQ: Performance IQ; ADHD: Attention deficit hyperactivity disorder; EEG: Electroencephalogram; CVS: Chorionic villi sampling.

## Competing interests

The authors declare that they have no competing interests.

## Authors' contributions

NT contributed medical and psychological data, treatment recommendations, edited and revised manuscript. SH provided input on genetics, etiology, and genetic counseling and revised manuscript. AS conducted literature review and contributed to medical description and differential diagnosis. RW contributed to review of psychological features and recommendations. LW initiated review, developed outline for manuscript with references. All authors read and approved the final manuscript.

## Consent

Written informed consent was obtained from the patients for publication and accompanying images. A copy of the written consent is available for review by the Editor-in-Chief of this journal.
